# Chronobiological changes due to school closures during the COVID-19 pandemic among adolescents in the DOrtmund Nutritional and Anthropometric Longitudinally Designed cohort study

**DOI:** 10.1007/s00431-023-04963-9

**Published:** 2023-04-10

**Authors:** Ines Perrar, Ute Alexy, Nicole Jankovic

**Affiliations:** 1 Institute of Nutritional and Food Sciences-Nutritional Epidemiology, DONALD Study, University of Bonn, Heinstück 11, 44225 Dortmund, Germany; 2 Institute of Nutritional and Food Sciences-Nutritional Epidemiology, University of Bonn, Friedrich-Hirzebruch-Allee 7, 53115 Bonn, Germany

**Keywords:** Chronobiology, Sleep, COVID-19, Adolescents, Social jetlag

## Abstract

Due to the lockdown of schools as one of the COVID-19 control measures, adolescents have had the opportunity to re-organise their daily lives; e.g. some of them have adapted their bedtimes to the new situation during the lockdown in favour of their own chronotype. Hence, we investigated differences in chronobiological characteristics (e.g., the midpoint of sleep, sleep duration or social jetlag (SJL); i.e., a discrepancy between biological and social timing) before and during the pandemic lockdown to examine potential changes. We asked participants from the ongoing open cohort Dortmund Nutritional and Anthropometric Longitudinally Designed (DONALD) study to fill out the Munich Chronotype Questionnaire during the COVID-19 lockdown and received the information of participants (*n* = 66) during the pandemic. A reference group matched for age, season, and sex was randomly selected from the DONALD study to assess participants’ chronobiological characteristics prior to (*n* = 132) the pandemic. Analyses of covariance were applied to examine differences between the two groups reflecting the situation before and during the COVID-19 pandemic. Participants were aged 9–18 years (52% males). In the current examination, average sleep duration across the week was higher among adolescents during the pandemic (*β* = 00:30; *p* = 0.0006) and social jetlag was significantly lower (*β* =  −00:39; *p* < 0.0001).

* Conclusion*: Our results showed that the COVID-19 lockdown enabled adolescents to adapt their sleeping habits according to their naturally late chronotype, which led to a significant reduction in SJL. These observations are likely to be explained by the effect of school closure.

**Table:** 

**What is Known:** *• Under ‘normal conditions’ without pandemic lockdowns, adolescents accumulate a lack of sleep due to social obligations such as school starts, resulting in social jetlag.* • *A late chronotype or exposure to social jetlag is a known risk factor for the development of chronic diseases.*	
**What is New:** *• The COVID-19 lockdown represents a ‘natural experiment’ that enables adolescents to adhere to their internal biological clock. Without the usual social obligations, social jetlag can be significantly reduced.*

**Supplementary Information:**

The online version contains supplementary material available at 10.1007/s00431-023-04963-9.

## Introduction

The COVID-19 pandemic has challenged the habitual lifestyle of children and adolescents all over the world. In Germany, the federal states opted for pandemic-related school closures repeatedly (first wave: 14.03.2020 to 11.08.2020; second wave: 14.12.2020 to 31.05.2021) to minimise the spread of the virus. During the school closures, children and adolescents were given tasks for home-schooling, and some schools offered the possibility to attend a few classes digitally. On the contrary, adolescents had the opportunity to organise their daily routines in a more self-determined way during school closures. Adolescents shift from an earlier to a later chronotype naturally [1]. Meaning, adolescents start sleeping later at night and prefer to sleep longer in the morning. This change may result in a larger social jetlag, i.e., a discrepancy between biological and social timing during schooldays [2]. However, adolescents may benefit from the COVID-19 lockdown, which may enable adherence to the inner biological rhythm instead of the social rhythm, as reported by other studies that relied on survey data [3]. Thus, we hypothesised that school closures have the potential to reduce social jetlag in free-living adolescents as they support living in accordance with the circadian rhythm of the individual chronotype.

Therefore, we aimed to compare chronobiological characteristics among adolescents (9–18 years) during school closures with pre-pandemic periods, using data from the Dortmund Nutritional and Anthropometric Longitudinally Designed (DONALD) study.

## Materials and methods

### Study design and population

The DONALD study is an ongoing cohort study, which started to collect data on diet, growth, development, and metabolism of healthy children and adolescents in Dortmund, Germany, and surrounding areas in 1985. Study design, objectives, inclusion criteria, and exclusion criteria have been described elsewhere [4]. The study design of the DONALD study was approved by the Ethics Committee of the University of Bonn (approval numbers: 098/06 and 185/20) with the ethical standards as laid down in the 1964 Declaration of Helsinki and its later amendments. All examinations are performed with parental consent and, from the age of 16 years onwards, on children’s written consent.

For the current analyses, we included 198 DONALD participants who provided Munich Chronotype Questionnaire (MCTQ) data. In total, 66 participants provided data during the pandemic (in 2020–2021) for whom we could define proper ‘references’ of 132 participants who provided data before the pandemic lockdown (2014–2019). As epidemiological data indicate a shift from an earlier to a later chronotype during adolescence [7], we decided not to use data from the same individuals before and during the pandemic. Instead, we selected a matched reference group by age, sex, and season [7], from the data pool of the DONALD study twice as large as the pandemic lockdown sample as mentioned above.

### Chronobiological data

The MCTQ is applied since 2014 in participants of the DONALD study starting at the 10-year visit. An English online version is publicly available (https://www.thewep.org/documentations/mctq/item/english-mctq-full-children). The MCTQ data allows the derivation of the following variables: midpoint of sleep (h:min) representing the person’s chronotype; average daily sleep duration on schooldays (h:min); average daily sleep duration on free days (h:min); average sleep duration across the week (h:min); social jetlag (h:min) [1]. Chronotype and social jetlag calculations are explained elsewhere [5]. To adapt the MCTQ to the situation of the pandemic lockdown, we re-formulated the MCTQ as follows: ‘Imagine a typical week of your sleep during the so-called lockdown in the period between March and June of 2020 and also the period between December 2020 and May 2021’. During the COVID-19 lockdown, the DONALD study centre continued to examine participants according to the study protocol. Hence, a total of 100 MCTQs could be collected during the COVID-19 lockdown period. All materials were sent back to the study centre by mail. Of those, 14 questionnaires had to be excluded due to incorrect data, and 20 participants had no proper reference partner; thus, the final sample had a total of 66 participants.

### Statistical analyses

Statistical analyses were performed using SAS^®^ procedures (version 9.4; Cary, NC, USA). The significance level was set to *p* < 0.01 to implement Bonferroni correction for the five chronobiological parameters analysed (0.05/5).

Sample characteristics in Tables 1 and S1 are presented as medians with their interquartile ranges for continuous variables or as frequencies and percentages for categorical variables. The average weekly sleep duration was compared to the recommendations of the American Academy of Sleep Medicine (AASM) [6]. In the main analyses, we compared chronobiological data before and during school closures using the analysis of covariance. Methods used to select confounding factors are described elsewhere [7]. Missing values (overweight status of the participant (*n* = 1), maternal overweight status (*n* = 4), maternal high educational status (*n* = 2), midpoint of sleep (*n* = 1) sleep duration on schooldays (*n* = 2), sleep duration on free days (*n* = 1), average sleep duration across the week (*n* = 3), and social jetlag (*n* = 3)) were imputed by the respective median of the total sample under the assumption that missing data are missing at random. Finally, we ran a sensitivity analysis on a complete-case sample without imputing missing data (data not shown).

**Table 1:** Chronobiological characteristics^a^ of DONALD participants (9–18 years) prior (*n* = 132) as well as during the COVID-19-related school closures (*n* = 66)

	**Prior pandemic**^**b**^	**Pandemic**^**c**^	**ANCOVA**^d^	
			***β*-estimate**	***p*-value**
**Chronobiological data**				
Midpoint of sleep^e^ (h:min)	4:00 (3:19; 04:40)	04:13 (03:30; 05:13)	00:12	0.1725
Sleep duration on schooldays (h:min)	7:50 (07:10; 8:45)	08:12 (07:10; 09:05)	00:15	0.1088
Sleep duration on free days (h:min)	9:24 (8:20; 10:10)	09:30 (08:30; 10:10)	00:08	0.5053
Sleep duration across the week (h:min)	08:17 (07:36; 08:59)	08:46 (08:06; 09:36)	00:30	0.0006*
Social Jetlag (h:min)	01:57 (01:25; 02:37)	1:10 (00:45; 02:10)	−00:39	< 0.0001*

^*^Significant *p*-values were marked with an asterisk

^a^Chracteristics are presented as medians (25th; 75th percentile)

^b^Years before the COVID-19 pandemic (2014–2019)

^c^School closures during the COVID-19 pandemic in 2020 and 2021 (15th March to 11th August 2020; 14th December 2020 to 31st May 2021) in Dortmund, Germany

^d^Statistic models for the ANCOVA were adjusted for sex (male, female), age (years), overweight status of the participant (yes, no), maternal data (overweight status (yes, no); high educational status (> 12 years; yes, no); employment (yes, no)) and season (spring, summer, autumn, winter)

^e^Midpoint of sleep (MFS) represents the underlying chronotype. Earlier MFS values represent an earlier chronotype and later MFS represents a later chronotype

## Results

We included a total of 198 participants aged 9–18 years. A randomly selected age-, sex-, and season-matched sample of 132 participants was included with pre-pandemic data, while 66 participants provided data during the pandemic (52% males). Maternal and anthropometric characteristics reflect a relatively high socioeconomic status of the participants (low overweight, < 35%; high education, > 82%, low unemployment, < 89%). During school closures, the average sleep duration across the week was significantly higher among adolescents (*β* = 00:30; *p* = 0.0006), and social jetlag was significantly lower (*β* =  −00:39; *p* < 0.0001) (Table 1).

For most participants (*n* = 110; 56%), the average sleep duration across the week during school closures met the AASM recommendations [6]. The percentage of participants who did not meet the AASM recommendations was higher before school closures (*n* = 69; 52%) than during the pandemic (*n* = 13; 29%).

## Discussion

In the current analysis, we investigated the impact of school closures during the COVID-19 pandemic lockdown on chronobiological characteristics among healthy adolescents by comparing data collected from participants during school closures with those collected from members of an age-, sex-, and season-matched reference group from the DONALD study. Our data suggest that the average sleep duration across the week was higher during school closures and the social jetlag of the participants was lower.

To our knowledge, only one other study [3] examined changes in chronobiological parameters due to school closures during the pandemic among adolescents. The Western-Norway Adolescent Longitudinal Sleep (WALOSS) study showed similar results to ours regarding social jetlag, which was significantly reduced from 2:37 in 2019 to 1:53 during school closures in 2020 among adolescents in Norway [3].

The usual early start of school lessons poses a challenge for adolescents, who naturally show a late chronotype [1], which has a direct impact on social jetlag. The COVID-19 pandemic offered adolescents the opportunity of a self-determined daily schedule, despite the obligation of home-schooling. Thus, in line with the underlying biology, our data indicate that natural sleep patterns adapted to the personal chronotype due to the biological clock during the pandemic; hence, the social jetlag was significantly reduced. The reduction of social jetlag is a positive change since living against the inner clock is discussed among other things to induce the risk of overweight and obesity [2, 5]. As mentioned earlier by Saxvig et al. [3], such results need to be treated with caution since the observed changes during the lockdown may not necessarily be comparable with changes due to a later school start under normal conditions.

As a limitation of the present study, no data on sleep quality or mental health were collected. However, the focus of the present analysis was on chronobiological parameters. Further limitations of the current study include the limited number of participants as well as the high socioeconomic status of the participants [4], which both limit the generalisability of our findings. Nevertheless, the average sleep durations across the week prior to and during the pandemic in the present study were similar to those reported in a large representative German survey [8]. The response rate for the MCTQ during lockdown was lower than that planned for the research project, probably due to social distancing and the challenge of coping with the new situation. Sensitivity analyses in which missing data were not imputed corroborated our results (data not shown). Although we can generally not rule out the fact that missing data are really missing at random [9], our data show no specific pattern of missing data.

All data were collected using the same methods before and during the pandemic. Also, the MCTQ was validated previously against actimetry and biochemical rhythms [1], as well as the Morningness–Eveningness Questionnaire [10], showing high correlations in both cases. The MCTQ has also been applied earlier among adolescents.

Overall, our findings suggest that healthy adolescents may quickly adapt their sleep durations to their own internal clock in a situation without strict social constraints such as the fixed early starts of schooldays. This pandemic, which induced social-life deregulation, also reduces the SJL and offers important insights for political measures that may help to reduce SJL outside of pandemic circumstances.

## Supplementary Information

Below is the link to the electronic supplementary material.

Supplementary file1 (DOCX 26 KB)

Supplementary file2 (DOCX 15 KB)

## Data Availability

Data of the DONALD study is available upon request to epi@uni-bonn.de.

## Abbreviations

AASM: American Academy of Sleep Medicine
ANCOVA: Analyses of covariance
BMI: Body mass index
DONALD: Dortmund Nutritional and Anthropometric Longitudinally Designed
MCTQ: Munich Chronotype Questionnaire
SJL: Social jetlag
WALOSS: Western-Norway Adolescent Longitudinal Sleep Study
