# Supplementary material for: Chronobiological changes due to school closures during the COVID-19 pandemic among adolescents in the DOrtmund Nutritional and Anthropometric Longitudinally Designed cohort study
Source: Eur J Pediatr. 2023 Apr 10;182(6):2801–5. doi: 10.1007/s00431-023-04963-9 (PMC10088594; doi:10.1007/s00431-023-04963-9)
Supplement: Supplementary file 1 — Supplementary file1 (DOCX 26 KB) [file 431_2023_4963_MOESM1_ESM.docx]

**Supplementary material to the Brief Report**

**Chronobiological changes due to school closures during the COVID-19 pandemic among adolescents in Germany**

**Ines Perrar^1,2^, Ute Alexy^1^, Nicole Jankovic^1^**

^1^Institute of Nutritional and Food Sciences-Nutritional Epidemiology, University of Bonn, DONALD study, Heinstück 11, 44225 Dortmund, Germany; [iperrar@uni-bonn.de](mailto:iperrar@uni-bonn.de) (https://orcid.org/0000-0002-2830-6322); [alexy@uni-bonn.de](mailto:alexy@uni-bonn.de) (https://orcid.org/0000-0002-1488-5175); [njankovi@uni-bonn.de](mailto:njankovi@uni-bonn.de) (<https://orcid.org/0000-0002-9235-5356>)

^2^Institute of Nutritional and Food Sciences-Nutritional Epidemiology, University of Bonn, Friedrich-Hirzebruch-Allee 7, 53115 Bonn, Germany; [iperrar@uni-bonn.de](mailto:iperrar@uni-bonn.de) (https://orcid.org/0000-0002-2830-6322)

**Corresponding author:**

Nicole Jankovic, Institute of Nutritional and Food Sciences-Nutritional Epidemiology, University of Bonn, DONALD study, Heinstück 11, 44225 Dortmund, Germany; Phone: +49 23179221034, Fax: +49 23179221033; E-mail: [njankovi@uni-bonn.de](mailto:njankovi@uni-bonn.de), ORCID: https://orcid.org/0000-0002-9235-5356

**Table S1:** Sample characteristics of DONALD participants (9-18 years) participants (9-18 years) prior (n=132) as well as during the COVID-19-related school closures (n=66)

|  | **Prior pandemic^1^** | **Pandemic^2^** |
| --- | --- | --- |
| **n** | 132 | 66 |
| **Females/males** [%] | 64 (49)/68 (52) | 32 (49)/34 (52) |
| **Age** [years] | 15.0 (12.0; 17.0) | 15.1 (12.1; 17.0) |
| **Anthropometric data** |  |  |
| BMI [kg/m²]^3^ | 19.2 (17.2; 22.3) | 19.2 (17.5; 20.7) |
| Overweight [%] | 22 (17) | 7 (11) |
| **Socioeconomic factors [%]** |  |  |
| Maternal overweight^4^ | 46 (35) | 20 (30) |
| Maternal high educational status^5^ | 108 (82) | 61 (92) |
| Maternal employment | 117 (89) | 60 (91) |
| **Season^6^ [%]** |  |  |
| Spring | 18 (14) | 9 (14) |
| Summer | 68 (52) | 34 (52) |
| Autumn | 0.0 | 0.0 |
| Winter | 46 (35) | 23 (35) |
| **Physical activity^7^**  Low  Moderate  High | 25 (30)  30 (36)  29 (35) | 17 (41)  12 (29)  13 (31) |

Values are frequencies (n (%)) or medians (25th; 75th percentile)
^1^years before the COVID-19 pandemic started (2014-2019)

^2^School closures during the COVID-19 pandemic in 2020 and 2021 (15^th^ March – 11^th^ August 2020; 14^th^ December 2020-31^st^ May 2021) in Dortmund, Germany

^3^Body Mass Index cutoff values for children and adolescents. Description of anthropometric measurements and used cut-offs can be found in [7].

^4^Body Mass Index > 25 kg/m²
^5^≥12 years of schooling

^6^Season when filling out the Munich Chronotype Questionnaire

^7^Tertiles of estimated daily energy expenditure; n=126 (42 observations during school closures; 84 observations prior pandemic)
