# Supplementary material for: Chronobiological changes due to school closures during the COVID-19 pandemic among adolescents in the DOrtmund Nutritional and Anthropometric Longitudinally Designed cohort study
Source: Eur J Pediatr. 2023 Apr 10;182(6):2801–5. doi: 10.1007/s00431-023-04963-9 (PMC10088594; doi:10.1007/s00431-023-04963-9)
Supplement: Supplementary file 2 — Supplementary file2 (DOCX 15 KB) [file 431_2023_4963_MOESM2_ESM.docx]

**Supplementary material to the Brief Report**

**Chronobiological changes due to school closures during the COVID-19 pandemic among adolescents in Germany**

**Ines Perrar^1,2^, Ute Alexy^1^, Nicole Jankovic^1^**

^1^Institute of Nutritional and Food Sciences-Nutritional Epidemiology, University of Bonn, DONALD study, Heinstück 11, 44225 Dortmund, Germany; [iperrar@uni-bonn.de](mailto:iperrar@uni-bonn.de) (https://orcid.org/0000-0002-2830-6322); [alexy@uni-bonn.de](mailto:alexy@uni-bonn.de) (https://orcid.org/0000-0002-1488-5175); [njankovi@uni-bonn.de](mailto:njankovi@uni-bonn.de) (<https://orcid.org/0000-0002-9235-5356>)

^2^Institute of Nutritional and Food Sciences-Nutritional Epidemiology, University of Bonn, Friedrich-Hirzebruch-Allee 7, 53115 Bonn, Germany; [iperrar@uni-bonn.de](mailto:iperrar@uni-bonn.de) (https://orcid.org/0000-0002-2830-6322)

**Corresponding author:**

Nicole Jankovic, Institute of Nutritional and Food Sciences-Nutritional Epidemiology, University of Bonn, DONALD study, Heinstück 11, 44225 Dortmund, Germany; Phone: +49 23179221034, Fax: +49 23179221033; E-mail: [njankovi@uni-bonn.de](mailto:njankovi@uni-bonn.de), ORCID: https://orcid.org/0000-0002-9235-5356

**Table S2:** Childcare characteristics of the DONALD participants (subgroup n=66) during pandemic lockdown^1^

| **Childcare characteristics** |  |
| --- | --- |
| **Females/males [%]** | 48/52 |
| **Age [years]** | 15.1 (12.1; 17.0) |
| **Type of childcare before pandemic^2^** |  |
| School | 65 (99) |
| Others e.g. family members | 1 (1) |
| **Childcare visit during pandemic^1,3^** |  |
| Yes  days per week  hours per week | 1 (1)  1 (1; 1)  6 (6; 6) |
| No | 65 (99) |
| **Home Office situation during pandemic^1^** |  |
| Mother worked in home office | 13 (20) |
| Father worked in home office | 13 (20) |
| Both parents worked in home office | 21 (32) |
| No parent worked in home office | 19 (29) |

Values are frequencies (n (%)) or medians (25th; 75th percentile)
^1^School closures during the COVID-19 pandemic in 2020 and 2021 (15th March – 11th August 2020; 14th December 2020-31th May 2021) in Dortmund, Germany

^2^years before the COVID-19 pandemic started (2014-2019)

^3^For parents from system-relevant professions, such as doctors or nurses it was possible to use a so-called “emergency childcare” for their children while kindergarten and schools were officially closed.
